# Autonomic Responses during Labor: Potential Implications for Takotsubo Syndrome

**DOI:** 10.3390/jcdd8110152

**Published:** 2021-11-07

**Authors:** Georgios E. Papadopoulos, Theoxaris I. Evaggelou, Errikos K. Moulias, Orestis Tsonis, Konstantinos C. Zekios, Dimitrios N. Nikas, Petros Tzimas, Minas Paschopoulos, Theofilos M. Kolettis

**Affiliations:** 1 1st Cardiology Department, University Hospital of Ioannina, 45500 Ioannina, Greece; georgios.e.papadopoulos@gmail.com (G.E.P.); zekioskostas@gmail.com (K.C.Z.); dimitrios.nikas@gmail.com (D.N.N.); 2 Department of Obstetrics & Gynecology, University Hospital of Ioannina, 45500 Ioannina, Greece; Ev.Charis@gmail.com (T.I.E.); errmoulias@gmail.com (E.K.M.); orestis.tsonis@gmail.com (O.T.); mpasxop@uoi.gr (M.P.); 3 Anesthesiology Department, University Hospital of Ioannina, 45500 Ioannina, Greece; petzimas@gmail.com

**Keywords:** sympathetic responses, vagal responses, cesarean delivery, vaginal delivery

## Abstract

Takotsubo syndrome is a serious complication of labor. Although the pathophysiologic role of excessive sympathetic activation is established in this process, concurrent vagal responses have not been adequately described. Moreover, it remains unclear whether autonomic activity depends on the mode of delivery. Here, we explored the hypothesis that the different management of cesarean and vaginal delivery may elicit diverse responses affecting both autonomic arms. For this aim, continuous electrocardiographic recording was performed in 20 women during labor, and non-invasive indices of sympathetic and vagal activity were compared between the two modes of delivery. We report sympathetic prevalence during cesarean delivery, caused by marked vagal withdrawal, whereas autonomic activity was rather stable during vaginal delivery. These differences may be attributed to the effects of anesthesia during cesarean delivery, along with the protective effects of oxytocin administration during vaginal delivery. Our results provide further insights on autonomic responses during labor that may prove useful in the prevention of complications, such as takotsubo syndrome.

## 1. Introduction

Labor elicits autonomic responses that regulate uterine contractions and local blood flow. During this process, excessive sympathetic activation has been increasingly reported, leading to maternal cardiac complications, such as takotsubo syndrome [1]. Although uncommon, its clinical manifestations resemble acute coronary syndromes and carry substantial morbidity and mortality. Most of the cases occur after cesarean delivery, but apparently only rarely after vaginal delivery. For instance, out of 16 cases of labor-induced takotsubo syndrome, 13 (81%) were associated with cesarean delivery, performed under neuraxial or general anesthesia [2]. Similarly, seven cases were identified after vaginal delivery in a recent systematic review of the literature, as opposed to 28 after cesarean section [3]. The explanation for this observation is uncertain, with differences in pharmacologic regimens brought forward as potential causative factors [4].

Regional ventricular wall motion abnormalities, unattributable to coronary artery disease, are the hallmark of takotsubo syndrome. In this process, the catecholamine surge caused by stress appears to play a central role [5]. A recent evaluation of triggering events highlights the need for a broader definition of stress, to include a wide variety of emotions, encompassing even feelings of extreme happiness [6]. In addition to sympathetic activation, vagal responses are considered a key-element of autonomic activity accompanying various emotions [7]. These considerations apply particularly to women in labor, a period characterized by mixed feelings of anxious coping and fear for potential complications, coupled with joy for the imminent birth [8]. However, little is known on maternal sympathetic and vagal activity during labor and their possible dependence on the mode of delivery. This knowledge is essential since it has the prospect of aiding the prompt management of excessive autonomic responses.

In the present study, we explored the hypothesis that the mode of delivery affects sympathetic and vagal responses. For this aim, we prospectively compared noninvasive autonomic indices during cesarean versus vaginal delivery in a cross-sectional study.

## 2. Materials and Methods

### 2.1. Patient Population

During the recruitment period (November 2020 to March 2021), elective admissions at the obstetrics department of our Institution were screened daily, and women with unremarkable past medical history were considered eligible for the study. Particular attention was paid to valvular heart disease, hypertension, and diabetes mellitus. Moreover, high-risk pregnancies such as diabetes, preeclampsia or eclampsia were excluded, as were women on β-adrenergic blockers or other cardio-active medications. The study protocol conforms to the principles described in the Declaration of Helsinki and was approved by the Institutional Review Board. All of the participants gave a written informed consent prior to the study entry.

The study population consisted of 20 successive women that fulfilled the entry criteria, allocated into two equally sized groups. This sample size provides over 90% power for detecting a (clinically meaningful) 30% difference in autonomic variables (assuming a two-tailed hypothesis). The mean age was comparable between the two groups, i.e., 32 ± 5 years in women undergoing vaginal delivery group and 33 ± 4 years in women undergoing planned cesarean delivery. The mode of delivery was based on clinical grounds and on patient preference. The electrocardiogram was continuously recorded with a Schiller Medilog AR12 Plus device (Jaken Medical Inc., CA, USA), ensuring good contact of the electrode-patches to the skin. The device was removed 30–45 min after delivery of the newborn and the placenta. After direct questioning on the following day, all of the participants reported the absence of psychological distress caused by the recording procedure.

### 2.2. Delivery

**Figure 1:**
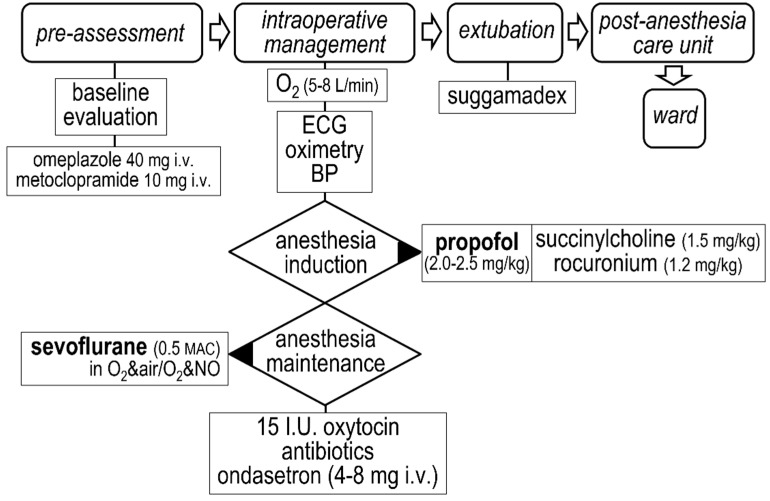
Cesarean delivery protocol at our Institution.

**Figure 2:**
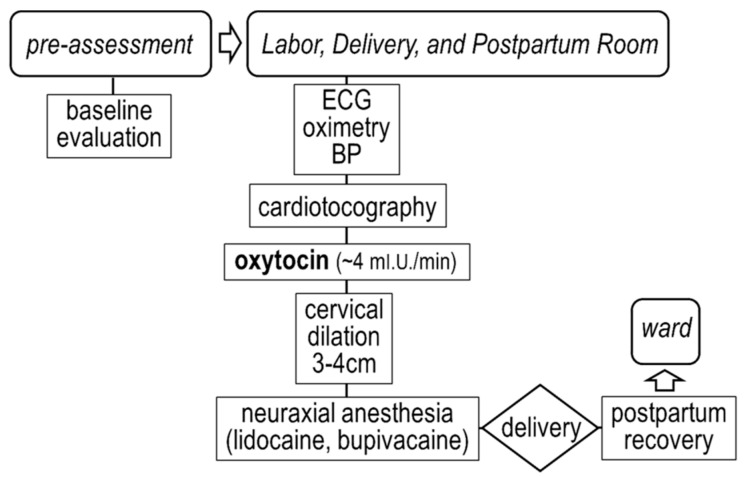
Vaginal delivery protocol at our Institution.

Delivery followed clinically indicated protocols at our Institution. In brief, the cesarean section was performed under general anesthesia, induced with propofol (2–2.5 mg kg^−1^) and maintained with sevoflurane (at 0.5 minimum alveolar concentration), as shown in Figure 1. Vaginal delivery, performed under neuraxial anesthesia, was preceded by continuous intravenous administration of oxytocin (~4 milli-units min^−1^), as shown in Figure 2.

### 2.3. Observation Time-Points

In view of previous data describing the onset of symptoms and signs of takotsubo syndrome shortly before or after delivery [2,3,4], we focused on the 1-h time-frame around delivery, by selecting the following time-points: 30 (t_−30_) and 15 (t_−15_) min prior to delivery, at delivery (t_0_), as well as 15 (t_+15_) and 30 (t_+30_) min after delivery. At each specific time-point, the reported values represent the average from the preceding 2-min recordings.

### 2.4. Heart Rate Variability Analysis

The analysis of the stored electrocardiograms was carried out with the use of the Medilog Darwin 2 software (Lifeline Medical Inc., FL, USA). We utilized indices derived from the heart rate variability analysis in the time- and frequency-domain. As no single variable offers a reliable description of each autonomic arm, particularly in short-term recordings [9,10], we opted for an integrated assessment using a combination of variables, describing separately sympathetic and vagal activity, as well as their balance [11].

### 2.5. Assessment of Sympathetic and Vagal Activity

Sympathetic activity was assessed with the low-frequency (LF) band (0.04–0.15 Hz) in the frequency-domain, expressed as the percent value of the total power and as the natural logarithm of the spectral power. The latter transformation was used to avoid a skewed distribution [12]. Vagal activity was assessed in the time-domain with the root mean square of successive differences between sinus inter-beat intervals (RMSSD) and with the percent of differences of adjacent sinus inter-beat intervals >50 msec (pNN50). In the frequency domain, we report the high-frequency (HF) band (0.15–0.40 Hz), expressed as the percent value of the total power and as the natural logarithm of the spectral power. As previously mentioned [10], the simultaneous presence of pNN50 < 3%, RMSSD < 25 ms, and heart rate > 65 beats per min was taken as an index of extremely low vagal activity.

In addition to heart rate, sympatho-vagal balance was assessed with the LF/HF ratio. Moreover, to overcome the limitations associated with the latter variable [13], we opted to include the standard deviation of consecutive sinus inter-beat intervals (SDNN) after the time-domain analysis. As both autonomic arms contribute to SDNN, it is considered of value in assessing their balance, particularly in short-term recordings [14].

### 2.6. Statistics

Variables, reported as the mean ± standard error of the mean, were assessed with the analysis of variance for repeated measures, and with *mode of delivery* and *time* as between- and within-groups factors, respectively. In the presence of variance, further comparisons were made with the post-hoc Duncan’s multi-stage test. Categorical variables were assessed with the χ^2^ test. For all of the comparisons, statistical significance was defined at *p* < 0.05.

## 3. Results

Labor and delivery were uneventful in all of the cases, with respect to women and fetuses. Continuous intravenous administration of oxytocin was commenced 6.2 ± 1.3 h prior to vaginal delivery, whereas the duration of cesarean delivery from the induction of anesthesia to extubation was 55 ± 4 min.

### 3.1. Sympatho-Vagal Balance

**Figure 3:**
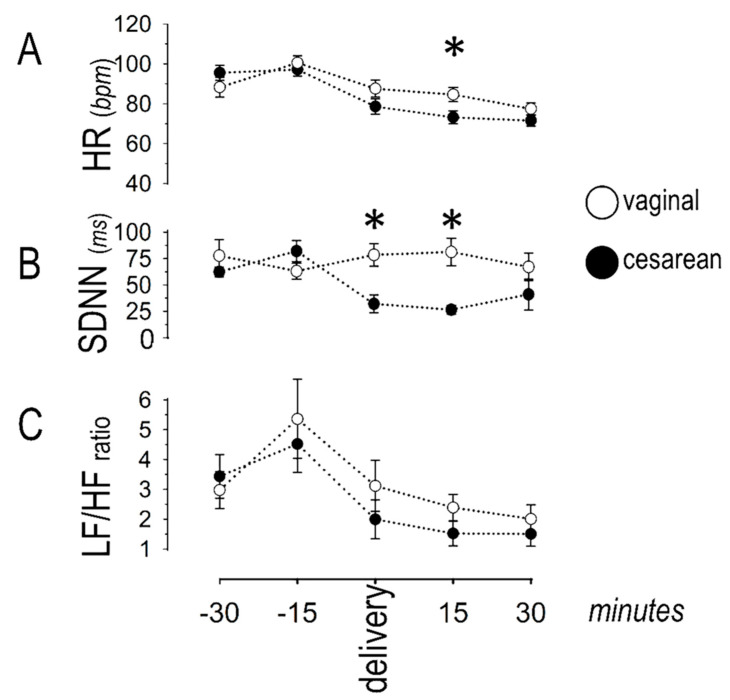
Sympatho-vagal balance; (**A**) except from the 15th minute post-delivery (asterisk), heart rate (HR, in beats per minute) was similar in the two groups; (**B**) the standard deviation of consecutive sinus inter-beat intervals (SDNN) was lower around cesarean delivery and 15 min thereafter (asterisks); (**C**) the low (LF) to high (HF) frequency band was similar in the two groups. Asterisks denote significant (*p* < 0.05) differences between groups.

Figure 3 depicts the temporal pattern of indices describing sympatho-vagal balance. Heart rate was similar in the two groups, except from the 15th-min post-delivery, when it was higher in the vaginal than in the cesarean group. No significant differences were observed between groups in the LF/HF ratio. During cesarean delivery, SDNN decreased over time, with lower values indicating vagal withdrawal. In this group, lower values were seen at t_0_, t_+15_, and t_+30_ (as compared to t_−30_), whereas no significant variance over time was noted during vaginal delivery. A between-groups comparison revealed lower SDNN during cesarean than during vaginal delivery at the time-points t_0_ and t_+15_.

### 3.2. Sympathetic Response

**Figure 4:**
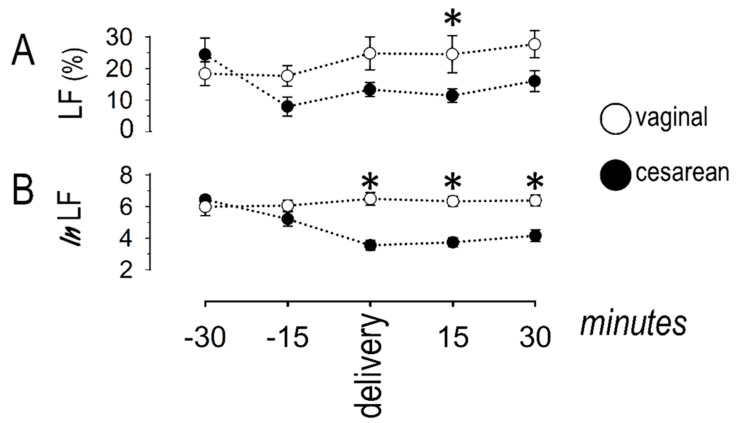
Sympathetic response, described by the low-frequency (LF) band in the frequency domain; (**A**) the percent (of the total) power was lower at the 15th minute post-cesarean delivery (asterisk); (**B**) the natural logarithm was lower around cesarean delivery as well as 15 and 30 min thereafter (asterisks). Asterisks denote significant (*p* < 0.05) differences between groups.

The power of the LF band in the frequency-domain analysis is depicted in Figure 4.

The LF band, expressed as the percent value of the total power or as the natural logarithm, remained stable along the observation period in women undergoing vaginal delivery. By contrast, the LF power decreased in women undergoing cesarean section, as compared to values at t_−30_. Specifically, the LF power (as percent of total power) decreased at t_−15_ and remained low in this group until t_+30_. The pattern of the natural logarithm of LF power was similar, with lower values recorded around cesarean delivery, persisting until the end of the observational period.

### 3.3. Vagal Response

**Figure 5:**
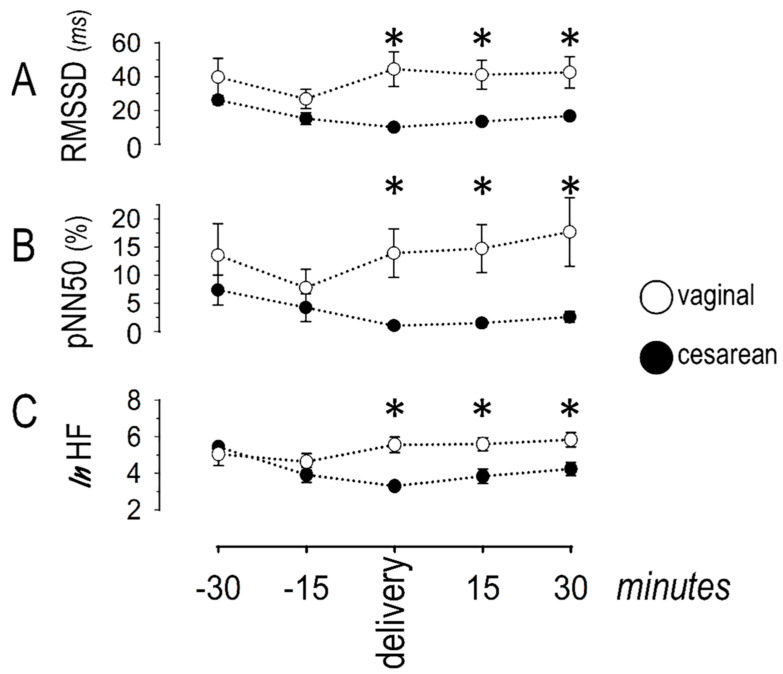
Vagal response, depicted as (**A**) the root mean square of successive differences between sinus inter-beat intervals (RMSSD); (**B**) the percent of differences of adjacent sinus inter-beat intervals >50 ms (pNN50); and (**C**) the natural logarithm of the high-frequency (HF) band in the frequency domain analysis. These variables indicate vagal withdrawal, evident around cesarean delivery, as well as 15 and 30 min thereafter (asterisks). Asterisks denote significant (*p* < 0.05) differences between groups.

The variables describing vagal response are shown in Figure 5.

**Figure 6:**
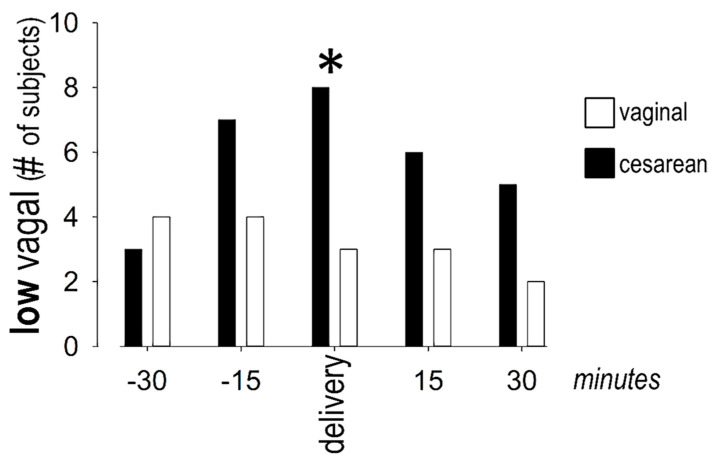
More women had extremely *low* vagal activity at the time of cesarean than vaginal delivery (asterisk, denoting significant (*p* < 0.05) difference between groups.).

During cesarean delivery, RMSSD decreased over time, indicating lower vagal activity. Specifically, lower values were seen at t_0_ and t_+15_, as compared to t_−30_. This trend was less pronounced during vaginal delivery, with differences seen only between t_−15_ and t_−30_. A between-groups comparison revealed lower RMSSD during cesarean than during vaginal delivery, at the time-points t_0_, t_+15_, and t_+30_. The pattern of pNN50 was similar, with decreased values (compared to t_−30_) observed at t_0_ during cesarean and at t_−15_ during vaginal delivery. A between-groups comparison revealed lower pNN50 during cesarean than during vaginal delivery at the time-points t_0_, t_+15_, and t_+30_. Finally, the natural logarithm of the HF power remained stable during vaginal delivery. By contrast, it decreased (from values at t_−30_) during cesarean delivery and remained low during the remaining period of observation. A between-groups comparison revealed lower values during cesarean delivery at the time-points t_0_, t_+15_, and t_+30_. More women had extremely low vagal activity (based on pNN50, RMSSD, and heart rate) around cesarean (8 out of 10) than around vaginal (3 out of 10) delivery (Figure 6).

## 4. Discussion

Autonomic responses during labor are clinically important, as they may herald takotsubo syndrome, a rare but serious condition. Hitherto research has focused on sympathetic activation, but less is known on concurrent vagal responses and their dependence on the mode of delivery. In the present study, we provide further insights, by comparing sympathetic and vagal activity between the two modes of delivery. Autonomic parameters were derived non-invasively, without psychological burden to the participants (imposed by the recording procedure) that could have interfered with the assessment. Our results were based on heart rate variability analysis in the time- and frequency-domain, using well-established methods [10].

### 4.1. Main Findings

We observed different responses between the two modes of delivery, affecting both autonomic arms. Compared to vaginal, cesarean delivery was associated with lower sympathetic activity, but was also characterized by prominent vagal withdrawal. As a result, the sympatho-vagal balance tilted towards sympathetic prevalence in this group, evident mainly around the time of delivery and persisting for the succeeding 15–30 min.

### 4.2. Autonomic Responses during Cesarean Delivery

We report lower sympathetic activity in women undergoing cesarean section, as compared to those undergoing vaginal delivery, in agreement with earlier findings of corresponding differences in maternal plasma norepinephrine levels [15]. More specifically, this study [15] reported lower norepinephrine levels after cesarean than after vaginal delivery (without oxytocin stimulation), whereas epinephrine levels were almost identical. An important additional finding in our study was the concurrent low vagal activity in this group, with 80% of women displaying marked vagal withdrawal (as opposed to only 30% of women undergoing vaginal delivery). These responses can be attributed to the previously described effects of propofol, used for the induction of general anesthesia in our study. In this regard, propofol has been previously shown to exert potent vagolytic effects, evidenced by reduced HF power in the range of 20–50%, depending on the depth of anesthesia [16]. These effects are thought to be mediated by lower systemic vascular resistance and hypotension, whereas the absence of reflex sympathetic activation can be explained by the simultaneous direct sympatholytic effects of propofol [17]. Moreover, sevoflurane, used for anesthesia maintenance in our population, exerts direct sympatholytic effects at multiple levels [18].

A noteworthy finding in our study was the poor correlation between autonomic alterations and heart rate. Based on the marked vagal withdrawal (leading to sympathetic prevalence) during cesarean delivery, heart rate, reflecting sympatho-vagal balance, would have been expected to be higher. However, this finding was not only absent, but, in fact, heart rate was lower at the 15th minute post-cesarean delivery. This observation can be attributed to the direct pharmacologic action of propofol on the sinus node, which accounts for its well-described bradycardic effects [19]. Despite the low dosages used only for anesthesia induction, these effects appeared long-lasting, most likely attributed to the relatively long half-life (40 min) of propofol [20].

### 4.3. Autonomic Responses during Vaginal Delivery

We report relatively stable indices of sympathetic and vagal activity during vaginal delivery, in keeping with previous reports [21,22]. In addition to the effects of analgesia, continuous oxytocin administration (for ~6 h prior to vaginal delivery) may have contributed to the observed autonomic responses in this group. Indeed, amidst the pleiotropic actions of this peptide-hormone, its anxiolytic properties [23] and the modulation of the autonomic nervous system have attracted substantial research interest [24]. Several lines of evidence indicate potent vagal activation by oxytocin [25], although the concurrent amelioration of sympathetic responses is still questionable. Moreover, differences in endogenous oxytocin have been reported between the two modes of delivery, likely caused by additional surges during vaginal delivery [26]. Finally, our findings may relate to the previously noted complex emotional profile of women in labor, characterized by disparate feelings of stress and satisfaction for a joyful event [8]. Furthermore, marked differences have been reported in the two modes of delivery, consisting of positive emotions immediately after vaginal delivery, as opposed to occasional disappointment and sense of failure after cesarean delivery [27]. Therefore, our study calls for additional investigation on the impact of emotions on the autonomic status during labor.

### 4.4. Implications for Takotsubo Syndrome

Our findings provide further insights on the pathophysiology of labor-induced takotsubo syndrome. The diverse responses, depending on the delivery mode, may aid in the explanation for the previously reported higher incidence during cesarean section [2,3,4]. Moreover, the swift autonomic alterations observed in the latter group are in keeping with clinical data indicating that the onset of takotsubo invariably occurs shortly after or even *during* surgery [2]. Therefore, a high index of suspicion should be exercised for early identification of excessive autonomic responses.

Our study provides support to the hypothesis of autonomic imbalance underlying takotsubo syndrome. In this regard, Akashi et al. [28] reported sympathetic activation and vagal withdrawal in 10 patients during the acute phase. This concept was further explored in a cohort of 10 women with remote history of takotsubo syndrome [29]. These patients displayed high sympathetic and low vagal responses during Valsalva maneuver and tilt testing, as well as during cognitive and emotional stimulation.

Contrary to prior understanding, rich cholinergic innervation has been demonstrated in the ventricular myocardium [30], although many aspects of its significance on electrical and mechanical function remain unclear. Nonetheless, earlier [31] and more recent [32] evidence indicates that regional vagal activation in the left ventricle not only modulates inotropic responses, but can also exert cardioprotective actions during myocardial ischemia. These actions appear to be mediated via suppression of ischemia-induced local norepinephrine release, as shown in an in vivo animal model, where interstitial norepinephrine levels were decreased by 70% after electrical stimulation of the vagus nerve [33]. Whether similar actions occur also during takotsubo syndrome remains to be examined in future research. To this end, the investigation of regional anatomic and/or functional differences in sympathetic and vagal nerve terminals is required, as a potential explanation for the corresponding wall motion abnormalities.

### 4.5. Implications for Other Clinical Conditions beyond Takotsubo Syndrome

Autonomic responses during delivery are clinically important in various other conditions. For instance, hypertrophic cardiomyopathy, increasingly diagnosed in pregnant women, is known to add a risk. Excessive sympathetic activation during delivery may trigger ventricular arrhythmias in this condition, whereas acute pulmonary edema may occur in the presence of severe left ventricular outflow tract obstruction [34]. In addition, mitral regurgitation, although invariably well tolerated, poses an increased risk during delivery, if complicated by left ventricular dysfunction or pulmonary hypertension [35].

### 4.6. Excessive Autonomic Changes Caused by Pharmacologic Agents

Our results emphasize the need for clinical assessment of the potential impact on the autonomic status of medications administered during labor. However, this task is often difficult, given the long list of candidate pharmacologic agents. For instance, potential triggers include medications used during neuraxial anesthesia, as well as sympathomimetic and vagolytic agents, antihistamines, tricyclic antidepressants, and levothyroxine [36]. The variety of agents commonly used for induction and maintenance of general anesthesia increases the complexity of this matter [37]. The proper management of each individual case is required, including prompt treatment when excessive sympathetic prevalence is suspected.

### 4.7. Strengths and Limitations

The present study offers a temporal description of sympathetic and vagal responses during labor, focusing on their dependence on the delivery mode. Our study is sufficiently powered for detecting clinically pertinent changes in autonomic variables. We focused on the time-frame around delivery, during which autonomic indices were reported at dense time-points, based on previous reports emphasizing the high rate of complications during this period [2,3,4]. The selection of this protocol is further supported by earlier clinical results, demonstrating a rapid (within minutes) decline of epinephrine levels after vaginal delivery, whereas norepinephrine rise displayed a more prolonged course [38]. Therefore, our results may have potential clinical implications in the prevention of complications during delivery.

Despite these merits, two limitations should be acknowledged: First, a larger cohort could have enabled the detection of more subtle, but still clinically relevant, changes in variables describing the autonomic status. Second, general anesthesia is part of the cesarean protocol in our Institution, based on data ascertaining its safety [39]. Nonetheless, our results cannot be extrapolated to caesarean section under neuraxial anesthesia, thus calling for further data in this regard.

## 5. Conclusions

In this prospective cross-sectional observational study, we examined autonomic activity during uncomplicated labor. We found disparate responses, depending on the mode of delivery, with vagal withdrawal characterizing cesarean delivery. Our study reinforces previous observations on the effects of anesthesia and draws attention to positive aspects of oxytocin administration. Further studies are expected to deepen our knowledge on the autonomic responses during labor.

## References

[1] Oindi F.M., Sequeira E., Sequeira H.R., Mutiso S.K. (2019). Takotsubo cardiomyopathy in pregnancy: A case report and literature review. BMC Pregnancy Childbirth.

[2] Minatoguchi M., Itakura A., Takagi E., Nishibayashi M., Kikuchi M., Ishihara O. (2014). Takotsubo cardiomyopathy after cesarean: A case report and published work review of pregnancy-related cases. J. Obstet. Gynaecol. Res..

[3] Citro R., Lyon A., Arbustini E., Bossone E., Piscione F., Templin C., Narula J. (2018). Takotsubo syndrome after cesarean section: Rare but possible. J. Am. Coll. Cardiol..

[4] Zdanowicz J.A., Utz A.C., Bernasconi I., Geier S., Corti R., Beinder E. (2011). “Broken heart” after cesarean delivery. Case report and review of literature. Arch. Gynecol. Obstet..

[5] Pelliccia F., Kaski J.C., Crea F., Camici P.G. (2017). Pathophysiology of takotsubo syndrome. Circulation.

[6] Ghadri J.R., Sarcon A., Diekmann J., Bataiosu D.R., Cammann V.L., Jurisic S., Napp L.C., Jaguszewski M., Scherff F., Brugger P. (2016). Happy heart syndrome: Role of positive emotional stress in takotsubo syndrome. Eur. Heart J..

[7] Pinna T., Edwards D.J. (2020). A Systematic review of associations between interoception, vagal tone and emotional regulation: Potential applications for mental health, wellbeing, psychological flexibility, and chronic conditions. Front. Psychol..

[8] Hauck Y., Fenwick J., Downie J., Butt J. (2007). The influence of childbirth expectations on Western Australian women’s perceptions of their birth experience. Midwifery.

[9] Nunan D., Sandercock G.R., Brodie D.A. (2010). A quantitative systematic review of normal values for short-term heart rate variability in healthy adults. Pacing Clin. Electrophysiol..

[10] Malik M., Bigger J.T., Camm A.J., Kleiger R.E., Malliani A., Moss A.J., Schwartz P.J. (1996). Heart rate variability. Standards of measurement, physiological interpretation, and clinical use. Task Force of the European Society of Cardiology and the North American Society of Pacing and Electrophysiology. Eur. Heart J..

[11] Shaffer F., Ginsberg J.P. (2017). An overview of heart rate variability metrics and norms. Front. Public Health.

[12] Kuo T.B., Lin T., Yang C.C., Li C.L., Chen C.F., Chou P. (1999). Effect of aging on gender differences in neural control of heart rate. Am. J. Physiol..

[13] Billman G.E. (2013). The LF/HF ratio does not accurately measure cardiac sympatho-vagal balance. Front. Physiol..

[14] Shaffer F., McCraty R., Zerr C.L. (2014). A healthy heart is not a metronome: An integrative review of the heart’s anatomy and heart rate variability. Front. Psychol..

[15] Jones III C.M., Greiss F.C. (1982). The effect of labor on maternal and fetal circulating catecholamines. Am. J. Obstet. Gynecol..

[16] Kanaya N., Hirata N., Kurosawa S., Nakayama M., Namiki A. (2003). Differential effects of propofol and sevoflurane on heart rate variability. Anesthesiology.

[17] Ebert T.J., Muzi M., Berens R., Goff D., Kampine J.P. (1992). Sympathetic responses to induction of anesthesia in humans with propofol or etomidate. Anesthesiology.

[18] Thorlacius K., Zhoujun C., Bodelsson M. (2003). Effects of sevoflurane on sympathetic neurotransmission in human omental arteries and veins. Br. J. Anaesth..

[19] Colson P., Barlet H., Roquefeuill B., Eledjam J.J. (1988). Mechanism of propofol bradycardia. Anesth. Analg..

[20] Tramer M.R., Moore R.A., McQuay H.J. (1997). Propofol and bradycardia: Causation, frequency and severity. Br. J. Anaesth..

[21] Suzuki N., Sugawara J., Kimura Y., Nagase S., Okamura K., Yaegashi N. (2012). Assessment of maternal heart-rate variability during labor using wavelet-based power spectral analysis. Gynecol. Obs. Investig..

[22] Reyes J.J., Pena M.A., Echeverria J.C., Garcia M.T., Ortiz M.R., Vargas C., Gonzalez-Camarena R. (2011). Short-term heart rate dynamics of women during labor. Annu. Int. Conf. IEEE Eng. Med. Biol. Soc..

[23] Heinrichs M., Baumgartner T., Kirschbaum C., Ehlert U. (2003). Social support and oxytocin interact to suppress cortisol and subjective responses to psychosocial stress. Biol. Psychiatry.

[24] Carter C.S., Kenkel W.M., MacLean E.L., Wilson S.R., Perkeybile A.M., Yee J.R., Ferris C.F., Nazarloo H.P., Porges S.W., Davis J.M. (2020). Is oxytocin “nature’s medicine”?. Pharm. Rev..

[25] Norman G.J., Cacioppo J.T., Morris J.S., Malarkey W.B., Berntson G.G., Devries A.C. (2011). Oxytocin increases autonomic cardiac control: Moderation by loneliness. Biol. Psychol..

[26] de Geest K., Thiery M., Piron-Possuyt G., Vanden Driessche R. (1985). Plasma oxytocin in human pregnancy and parturition. J. Perinat. Med..

[27] Kjerulff K.H., Brubaker L.H. (2018). New mothers’ feelings of disappointment and failure after cesarean delivery. Birth.

[28] Akashi Y.J., Barbaro G., Sakurai T., Nakazawa K., Miyake F. (2007). Cardiac autonomic imbalance in patients with reversible ventricular dysfunction takotsubo cardiomyopathy. J. Assoc. Physicians.

[29] Norcliffe-Kaufmann L., Kaufmann H., Martinez J., Katz S.D., Tully L., Reynolds H.R. (2016). Autonomic findings in takotsubo cardiomyopathy. Am. J. Cardiol..

[30] Pauza D.H., Skripka V., Pauziene N., Stropus R. (2000). Morphology, distribution, and variability of the epicardiac neural ganglionated subplexuses in the human heart. Anat. Rec..

[31] Landzberg J.S., Parker J.D., Gauthier D.F., Colucci W.S. (1994). Effects of intracoronary acetylcholine and atropine on basal and dobutamine-stimulated left ventricular contractility. Circulation.

[32] Mastitskaya S., Marina N., Gourine A., Gilbey M.P., Spyer K.M., Teschemacher A.G., Kasparov S., Trapp S., Ackland G.L., Gourine A.V. (2012). Cardioprotection evoked by remote ischaemic preconditioning is critically dependent on the activity of vagal pre-ganglionic neurones. Cardiovasc. Res..

[33] Kawada T., Yamazaki T., Akiyama T., Li M., Ariumi H., Mori H., Sunagawa K., Sugimachi M. (2006). Vagal stimulation suppresses ischemia-induced myocardial interstitial norepinephrine release. Life Sci..

[34] L’Ecuyer E., Codsi E., Mongeon F.P., Dore A., Morin F., Leduc L. (2021). Perinatal and cardiac outcomes of women with hypertrophic cardiomyopathy. J. Matern. Fetal Neonatal Med..

[35] Tsiaras S., Poppas A. (2009). Mitral valve disease in pregnancy: Outcomes and management. Obstet. Med..

[36] Amariles P. (2011). A comprehensive literature search: Drugs as possible triggers of Takotsubo cardiomyopathy. Curr. Clin. Pharm..

[37] Littlejohn F.C., Syed O., Ornstein E., Connolly E.S., Heyer E.J. (2008). Takotsubo cardiomyopathy associated with anesthesia: Three case reports. Cases J..

[38] Lederman R.P., McCann D.S., Work B., Huber M.J. (1977). Endogenous plasma epinephrine and norepinephrine in last-trimester pregnancy and labor. Am. J. Obstet. Gynecol..

[39] Hawkins J.L., Chang J., Palmer S.K., Gibbs C.P., Callaghan W.M. (2011). Anesthesia-related maternal mortality in the United States: 1979–2002. Obs. Gynecol..

